# All-Cause Mortality and Incidence of Major Adverse Cardiac Events in Sickle Cell Nephropathy: A Comparative Study

**DOI:** 10.7759/cureus.15059

**Published:** 2021-05-16

**Authors:** Kunjan Udani, Nayda Parisio-Poldiak, Julia Campbell, Victor Collier, Pooja Patel

**Affiliations:** 1 Internal Medicine, Grand Strand Medical Center, Myrtle Beach, USA; 2 Medicine, Grand Strand Medical Center, Myrtle Beach, USA; 3 Medicine, Edward Via College of Osteopathic Medicine, Spartanburg, USA

**Keywords:** major adverse cardiac event, mace, sickle cell nephropathy, sickle cell disease, all-cause mortality

## Abstract

Background

Sickle cell disease (SCD) is an autosomal recessive disease resulting in hemolytic anemia and recurrent vaso-occlusive events. Consequently, it can result in a broad range of functional and structural renal and cardiac alterations. Chronic kidney disease (CKD), in SCD, is associated with proteinuria, microalbuminuria, and hemoglobinuria. Cardiac complications in SCD include pulmonary hypertension, left ventricular diastolic heart disease, dysrhythmia, and sudden death. In patients with advancing age, cardio-renal dysfunction can have substantial effects on morbidity and mortality. Our primary aim was to compare the incidence of major adverse cardiac events (MACE) and all-cause mortality in sickle cell nephropathy (SCN).

Methods

In this retrospective study, we used International Classification of Diseases (ICD)-10 codes to identify admissions in 2019 with a diagnosis of MACE with a prior diagnosis of SCD and/or SCN. Our search of the HCA Healthcare Enterprise Data Warehouse for adult patients >18 years yielded 6,693 patients with SCD, of which 658 patients (9.8%) had SCN. Primary endpoints were incidence of MACE and all-cause mortality. Patients with MACE encompassed those with nonfatal stroke, nonfatal myocardial infarction, and congestive heart failure (CHF) exacerbations. A secondary endpoint was length of stay (LOS). Logistic regression analysis was used for MACE and all-cause mortality. LOS was analyzed using multiple linear regression analysis. Results were considered statistically significant for analyses showing p <0.05. All outcomes were adjusted for demographic variables and comorbidities.

Results

Logistic regression, after adjustment for comorbidities, demonstrated that SCN patients had significantly higher odds of all-cause mortality (odds ratio [OR] 2.343, p = 0.035, 95% confidence interval [CI] 1.063-5.166) compared to patients without SCN. Compared to those without SCN, those with SCN did not have a higher odds of MACE (OR 1.281, p = 0.265, 95% CI 0.828-1.982). Linear regression for LOS did not reveal a significant association with SCN (p = 0.169, 95% CI 0.157-0.899).

Conclusion

Based on the analysis of 6,693 patients with SCD, SCN was associated with significantly higher odds of all-cause mortality. SCN was not associated with significantly higher odds of MACE or prolonged LOS.

## Introduction

Sickle cell disease (SCD) is a hematologic disorder caused by a single base-pair mutation in the gene for the beta-globin chain of adult hemoglobin (HbS) [1]. The disease is characterized by sickle shaped erythrocytes induced by tissue hypoxia or dehydration, leading to vaso-occlusion and hemolytic anemia [2].

Sickle cell patients with severe hemolytic anemia are predisposed to numerous complications such as pulmonary hypertension, ischemic stroke, recurrent infection, acute chest syndrome, deep vein thrombosis, pulmonary embolism, skin ulcers, and impaired vasomotor control [3,4]. Left ventricular diastolic dysfunction, elevated serum levels of N-terminal pro-B-type natriuretic peptide (NT-proBNP), hemoglobinuria, proteinuria, macro-albuminuria, chronic kidney disease (CKD), and coronary disease are all “biomarkers” that have been shown to help identify adult patients with SCD who are at a higher risk of morbidity and death [5].

The rich vascularity in the kidney coupled with low oxygen tension may make it more susceptible to sickle cell damage, leading to devastating effects, which include sickle cell-induced renal failure and sickle cell nephropathy (SCN) [2]. Microalbuminuria, an early manifestation of SCN, is prevalent in nearly 60% of patients aged >45 years but only 4%-12% of patients will develop serious, life-threatening end-stage renal disease [6]. Serial monitoring of the estimated glomerular filtration rate (eGFR) allows for evaluation of the progression of SCN. Rapid decline in eGFR has been associated with higher systolic blood pressure and proteinuria [7].

Cardiovascular disease was the most common cause of death in SCD patients from 1999-2009 [8,9,10]. Major adverse cardiac events (MACE) associated with SCD include cardiomyopathy, hypertension, heart failure, myocardial infarction, arrhythmias, and sudden death [11,12]. The spectrum of functional and structural renal and cardiac alterations in sickle cell hemoglobinopathy is broad; however, morbidity and mortality from end-organ dysfunction, especially cardio-renal dysfunction, are substantial [13].

SCD patients have elevated levels of systemic inflammatory biomarkers that suggest a tendency towards endothelial dysfunction, cardiovascular and thromboembolic risk [14]. CKD is a well-defined risk factor for cardiovascular events, but the characterization of a potential additional cardiovascular risk in the population with SCD and CKD has not been defined [15]. This study aims to compare all-cause mortality and incidence of MACE in SCN.

## Materials and methods

In this retrospective study, we evaluated adult patients (age ≥18 years) hospitalized with a diagnosis of MACE with a prior diagnosis of SCD and SCN during 2019 (n = 6,693). To acquire the dataset, we used International Classification of Diseases (ICD)-10 codes to query the HCA Healthcare Enterprise Data Warehouse, which encompasses 186 hospitals in 21 states across the United States. Patients younger than 18 years or undergoing outpatient surgery were excluded. This study received an institutional IRB exempt determination (2019-517).

Data were analyzed using SAS 9.4 (SAS Institute, Cary, NC) and IBM Statistical Package for Social Sciences (SPSS), version 24 (IBM Corp., Armonk, NY). Primary endpoints were incidence of MACE and all-cause mortality. Patients with MACE encompassed those with nonfatal stroke, nonfatal myocardial infarction, and congestive heart failure (CHF) exacerbations. A secondary endpoint was length of stay (LOS). Multivariate logistic regression was used to assess the relationships between MACE, CHF, and mortality. Multiple linear regression analysis was used to assess relationships with LOS. All outcomes were adjusted for demographic variables and comorbidities. An alpha level of 0.05, with possible correction for multiple comparisons, was used for type I error rate.

## Results

**Figure 1:**
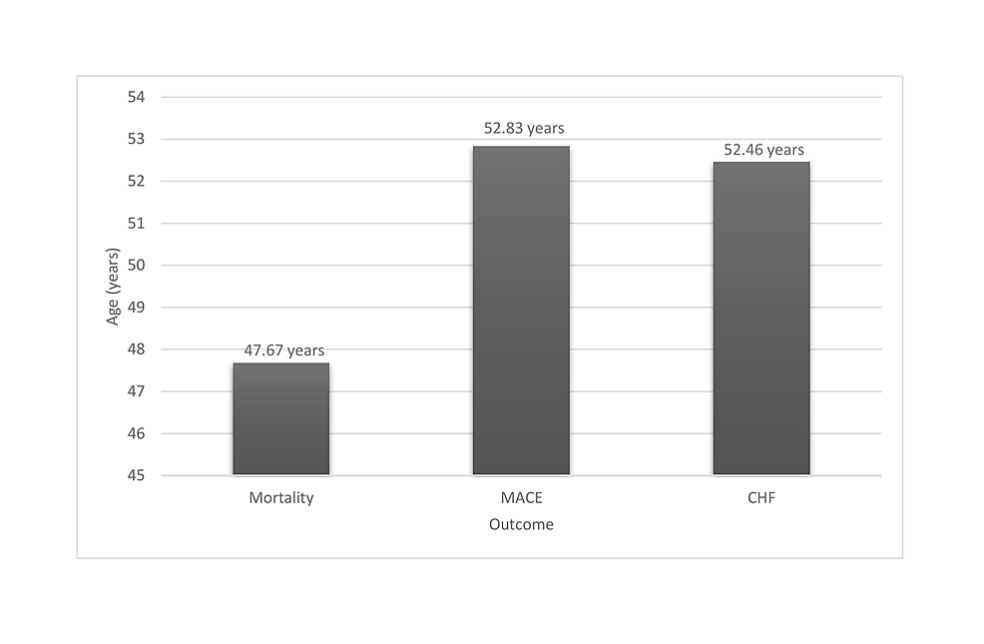
Sickle cell disease and outcome variable MACE: major adverse cardiac events, CHF: congestive heart failure

The study cohort included 6,693 adults patients identified with SCD with an average age of 36.34 ± 13.8 years. Table 1 summarizes the demographics of patients with SCD and related comorbidities. Patients were more likely to be African American (90.2%) and female (65.1%). 9.8% of the cohort had SCN, 3.6% had MACE, 12.8% had asthma, 9.1% were diabetic and 11% were tobacco users. The average age for patients who died in the hospital was 47.67 years, while the average for patients diagnosed with MACE and CHF were 52.83 and 52.46 years, respectively (Figure 1).

**Table 1:** Demographics of patients with sickle cell disease and related comorbidities MACE: major adverse cardiac events, COPD: chronic obstructive pulmonary disease,

Sickle Cell Disease (n = 6,693)	Number of Patients	Percentage
Sex		
Male	2337	34.9
Female	4356	65.1
Race		
Black	6037	90.2
White	295	4.4
Other	361	5.4
Cardiovascular		
Coronary Disease	245	3.6
MACE	242	3.6
Congestive Heart Failure	222	3.3
Peripheral Vascular Disease	35	0.5
Myocardial Infarction	22	0.3
Cardiac Arrest	20	0.2
Ventricular Arrhythmia	20	0.2
Cardiogenic Shock	11	0.1
Stroke	4	0.05
Pulmonary		
Asthma	859	12.8
Pulmonary Embolism	62	0.9
Acute Respiratory Failure	43	0.6
COPD	14	0.2
Renal		
Nephropathy	658	9.8
Chronic Kidney Disease	544	8.1
Acute Renal Failure	413	6.1
Proteinuria	61	0.9
Hemoglobinuria	1	0.01
GI/Liver		
Noninfectious Hepatitis	81	1.2
Acute Liver Failure	15	0.2
Neuro		
Transient Ischemic Attack	16	0.2
Endocrine		
Diabetes	611	9.1
Obesity	555	8.2
Thyrotoxicosis	23	0.3
Shock	10	0.1
Other		
Tobacco Abuse	740	11.0
Recreational Drug Use	430	6.4
Cannabis	230	3.4
Opioid	153	2.2
Cocaine	79	1.1
Alcohol Abuse	40	0.5
Amphetamine	25	0.3

**Figure 2:**
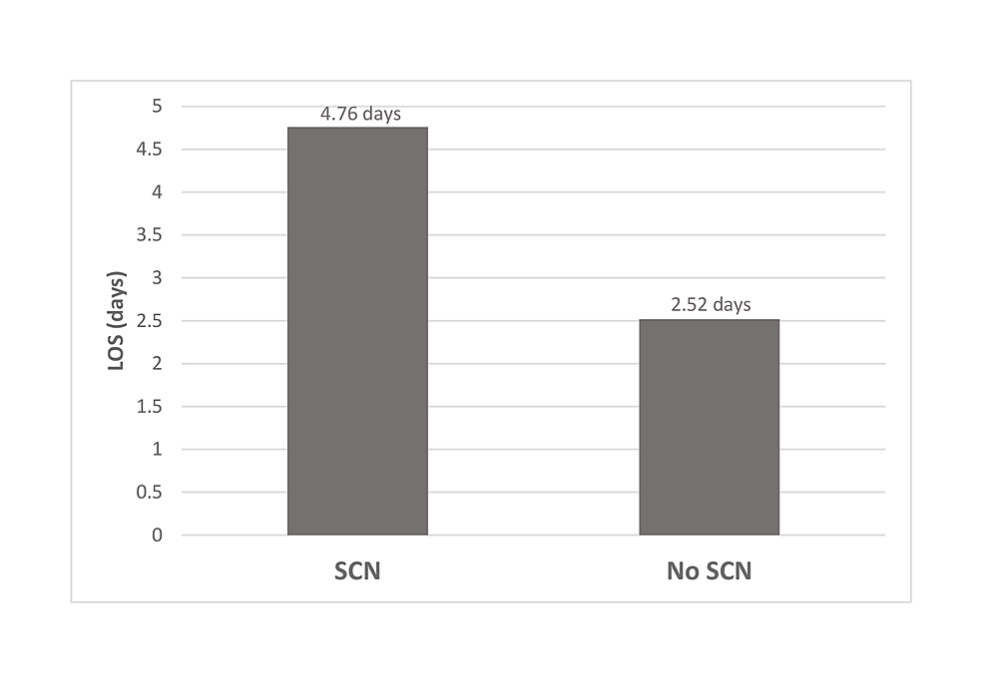
Length of stay of patients with and without sickle cell nephropathy LOS: length of stay, SCN: sickle cell nephropathy

SCN patients had an average LOS of 4.76 days compared to 2.52 days for sickle cell patients without nephropathy (Figure 2). On multivariate linear regression, SCN patients had a 0.371-day longer stay compared to patients without nephropathy, which did not yield a significant relationship between LOS and SCN (p = 0.169, 95% confidence interval (CI) (-0.157, 0.899)).

As seen in Table 2, patients with SCN had a significantly higher mortality [odds ratio (OR) 2.343 p = 0.035, 95% CI (1.063, 5.166)] compared with patients without nephropathy. There was trend toward an increased risk of CHF exacerbations [OR 1.438, p = 0.109, 95% CI (.923, 2.241)], but the OR for MACE events was not significant [OR 1.281, p = 0.265, 95% CI (.828, 1.982)], nor was the regression for LOS (p = 0.169, 95% CI (-.157, .899)).

**Table 2:** Odds ratio for mortality, CHF and MACE comparing patients with and without sickle cell nephropathy OR: odds ratio, CI: confidence interval, CHF: congestive heart failure, MACE: major adverse cardiac events

Nephropathy outcome	OR	P value	95% CI
Mortality	2.343	.035	1.063 - 5.166
CHF	1.438	.109	0.923 - 2.241
MACE	1.281	.265	0.828 - 1.982

## Discussion

This retrospective cohort study compared the incidence of MACE and all-cause mortality in patients with SCN. The key findings show that patients with nephropathy did not have a significant increase in MACE; however, these same patients had a significant increase in mortality, such that they were 2.3 times more likely to die when compared to patients without nephropathy. It has been well established in the literature that repeated vaso-occlusive crises are responsible for SCN that may range from renal dysfunction to CKD [2]. In addition, an association has been suggested between glomerulosclerosis and atherosclerosis [16]. The pathogenic process of oxidative stress atherosclerosis augments the progression of activated macrophages with foam cell formation that contribute to both the progression of glomerulosclerosis and atherosclerosis [17]. Evidence from transgenic, sickle cell mice supports the association between progression of renal disease and cardiovascular disease due to nitric oxide-mediated oxidant stress injury [18]. These data suggest that preventive strategies for cardiovascular disease and atherosclerosis may prevent the progression of kidney disease.

While subclinical renal abnormalities begin at an earlier age, end-stage renal disease in SCN usually occurs between the third and fifth decades of life [19,20]. Platt et al. reported an 18% mortality for SCD patients with organ dysfunction, particularly renal dysfunction. Notable predictors of mortality include progression of renal disease, severe anemia, seizures, acute chest syndrome, and more symptomatic disease [21]. Similarly, Hamideh and Alvarez reported mortality in 16.4% of patients with renal disease [10]. In a prospective study following 105 patients over five years, Galloway-Blake et al. reported a 38% mortality rate (40 out of 105 patients) for patients with SCD [22]. In our study of 6,693 SCD patients, we found an increased OR for mortality, which agrees with the aforementioned studies. We found that the average age for mortality was 47.67 years (Figure 1). This roughly agrees with the previously published data, which noted an average age of death at 35-44 years for males and 45-54 years for females [10,21].

Sickled erythrocytes adhere to vascular endothelium, resulting in a pro-inflammatory state, with subsequent platelet aggregation and vasoconstriction. This leads to thrombi formation in cerebral and cardiac blood vessels, causing strokes and myocardial infarction [23,24]. Cardiac blood supply could be compromised during sickling crises, causing hypoxia as well as myocardial ischemia. Recurrent myocardial ischemia often leads to CHF [25]. Additionally, chronic anemia due to SCD causes left ventricular dilation, eccentric hypertrophy, and diastolic dysfunction to maintain adequate cardiac output [5,12]. We found a significantly increased risk of CHF exacerbations, thus confirming that SCD plays a key role in the development of CHF.

When examining the impact of SCN on the duration of hospitalization, we did not observe a significant association between SCN and LOS. On the contrary, Yeruva et al. observed a 1.6-day increase in LOS for SCD patients diagnosed with CKD and 3.2 days for acute renal failure (ARF) [26]. In present-day medicine, LOS is a relevant topic due to the importance given to cost-effective high-value care. The chronicity of renal disease in SCD raises concern for high economic burden [27-29].

Our study has some potential limitations. Patients were identified using discharge ICD-10 codes through an electronic administrative database. The accuracy of the ICD-10 codes is influenced by several factors, including quality of communication between physicians and patients, clinicians’ expertise and precision of the diagnoses in medical records as well as coders’ experience and attention to choosing the best code. Hence, as an administrative database, the HCA Healthcare Electronic Data Warehouse may have variations in the degree of detail and accuracy. Additionally, this study had an intrinsic limitation for having a retrospective design with only one year of data collection, which may have affected its results.

## Conclusions

Based on the analysis of 6,693 patients with SCD, SCN was associated with significantly higher odds of all-cause mortality. SCN was not associated with significantly higher odds of MACE or prolonged LOS. While SCN may be associated with all-cause mortality, further studies are warranted to understand the association between SCN and cardiovascular complications.
